# Supplementary material for: Analysis of social interactions and risk factors relevant to the spread of infectious diseases at hospitals and nursing homes
Source: PLoS One. 2021 Sep 20;16(9):e0257684. doi: 10.1371/journal.pone.0257684 (PMC8452062; doi:10.1371/journal.pone.0257684)
Supplement: S1 File — Complete list of locations where sensors where placed. (PDF) [file pone.0257684.s001.pdf]

Locations of sensors

Hospital Here we present the rooms in the hospital:

- Clean Rinsing Room
- Patient Room
- Medication Room
- Staff Toilets
- Dirty Rinsing Room
- Patient Toilet
- Dirty storeroom
- Clean storeroom
- Hallway
- Office
- Lounge
- Staff-Room
- Meeting Room
- Kitchen
- Storeroom.

Nursing home Here we present the rooms in the nursing home:

- Apartment Room
- Patient Room
- Nursing Home Flat,
- Hallway
- Staff Toilet
- Kitchen
- Dirty Storeroom
- lounge
- Patient toilet
