# Supplementary material for: Analysis of social interactions and risk factors relevant to the spread of infectious diseases at hospitals and nursing homes
Source: PLoS One. 2021 Sep 20;16(9):e0257684. doi: 10.1371/journal.pone.0257684 (PMC8452062; doi:10.1371/journal.pone.0257684)
Supplement: S2 File — Brief description of practices for contact tracing at the time of publication. (PDF) [file pone.0257684.s002.pdf]

Current practice of contact tracing

We here provide a short description of the current methods for performing contact tracing at hospitals. Currently, there exist guidelines for the following two situations:

1. An HCW experiences symptoms for SARS-CoV-2.
2. An HCW is tested positive for SARS-CoV-2 based on a routine test.

If an HCW experiences symptoms The HCW needs to self-isolate themselves at once. On the next day have a test for SARS-CoV-2 conducted. On the third day, the answer to the test is available. If the test is positive, management at the hospital is informed and manually start performing contact tracing for the 48 hours before the first day of symptoms. In practice, they check shift schedules and manually interview the infected HCW. The HCW try to recall if they were wearing ICP equipment for all the encounters they have had. Personnel, identified as close contacts to the infected HCW, needs to be tested on day four and six. The personnel can return to work after the second negative test.

If an HCW is tested positive based on a routine test The HCW is sent home from work immediately. Management is informed and manually starts contact tracing for the last 48 hours in the same way as above. Personnel, identified as close contacts to the infected HCW, needs to be tested on day four and six. The personnel can return to work after the second negative test.
